# Resource-efficient bio-inspired visual processing on the hexapod walking robot HECTOR

**DOI:** 10.1371/journal.pone.0230620

**Published:** 2020-04-01

**Authors:** Hanno Gerd Meyer, Daniel Klimeck, Jan Paskarbeit, Ulrich Rückert, Martin Egelhaaf, Mario Porrmann, Axel Schneider

**Affiliations:** 1 Research Group Biomechatronics, CITEC, Bielefeld University, Bielefeld, Germany; 2 Department of Neurobiology and CITEC, Bielefeld University, Bielefeld, Germany; 3 Cognitronics and Sensor Systems Group, CITEC, Bielefeld University, Bielefeld, Germany; 4 Biomechatronics and Embedded Systems Group, Faculty of Engineering and Mathematics, University of Applied Sciences, Bielefeld, Germany; 5 Computer Engineering Group, Osnabrück University, Osnabrück, Germany; Durham University, UNITED KINGDOM

## Abstract

Emulating the highly resource-efficient processing of visual motion information in the brain of flying insects, a bio-inspired controller for collision avoidance and navigation was implemented on a novel, integrated System-on-Chip-based hardware module. The hardware module is used to control visually-guided navigation behavior of the stick insect-like hexapod robot HECTOR. By leveraging highly parallelized bio-inspired algorithms to extract nearness information from visual motion in dynamically reconfigurable logic, HECTOR is able to navigate to predefined goal positions without colliding with obstacles. The system drastically outperforms CPU- and graphics card-based implementations in terms of speed and resource efficiency, making it suitable to be also placed on fast moving robots, such as flying drones.

## Introduction

A prerequisite for autonomous behavior in mobile robots is the ability to navigate in cluttered terrain without colliding with obstacles. This ability requires an agent to process sensory information in order to generate appropriate motor commands. Nowadays autonomous mobile robots rely on active sensors (e.g. laser range finders, [1]) or extensive computations (e.g. Lucas-Kanade optic flow computation, [2]) to acquire and process relevant environmental information. Insects—despite their relatively small body size—show a remarkable behavioral performance when navigating in cluttered environments with minimal energy and computational expenditure. It seems plausible, that the application of these navigational abilities on a mobile robot also requires a resource-efficient approach. An important source of information for navigation is the extraction of visual motion cues from *optic flow* (i.e. the field of retinal image velocities during ego-motion or motion of objects in the environment), as it provides an agent with information about *self-motion*, *moving objects*, and also about the *three-dimensional structure of the environment* [3]. In flying insects the processing of optic flow has been shown to be relevant in the control of *flight stabilization*, *object detection*, *visual odometry* and *spatial navigation* [4–7]. From an engineer’s perspective the underlying mechanisms for processing optic flow are of great interest as they might help to reduce energy expenditure and to overcome computational restrictions of conventional approaches in robotic vision and behavioral control [8].

**Fig 1:**
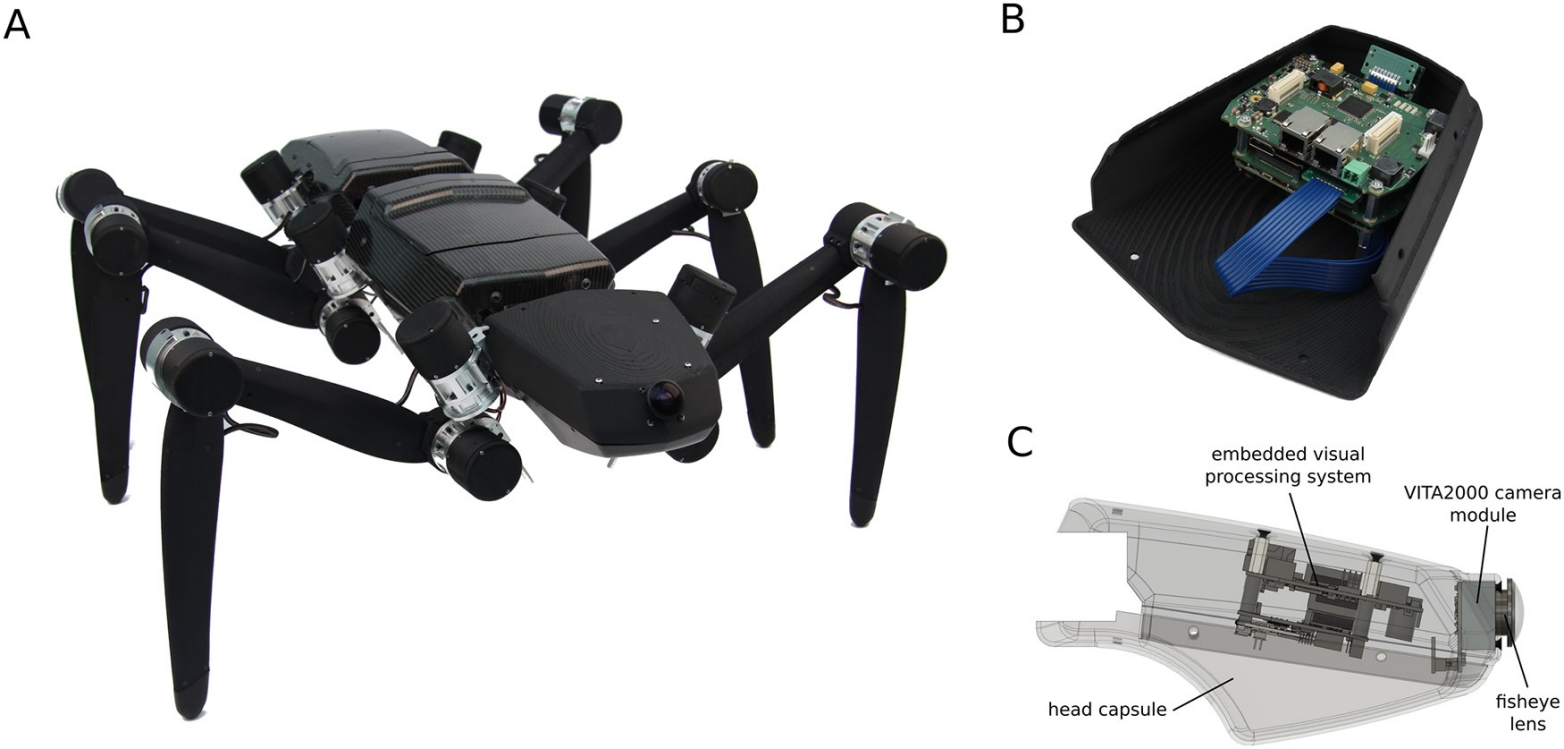
**(A)** The hexapod walking robot HECTOR is inspired by the stick insect *Carausius morosus*. For its design, the relative positions of the legs as well as the orientation of the legs’ joint axes have been adopted. The size of the robot has been scaled up by a factor of 20 as compared to the biological example which results in an overall length of roughly 0.9 m. All 18 drives for the joints of the six legs are serial elastic actuators. The mechanical compliance of the drives is achieved by an integrated, sensorized elastomer coupling. The bio-inspired control of walking is achieved via a conversion of the WALKNET approach. Bottom view **(B)** and rendered side view **(C)** of the front segment of HECTOR. The upper compartment of HECTOR’s front segment has been equipped with a *panoramic camera system* and an *embedded hardware module* for processing of visual information. This allows the robot to perform visually-guided tasks such as collision avoidance or navigation.

In this study, a highly resource- and energy-efficient embedded hardware platform is presented which was specifically designed to perform insect-inspired visual processing on autonomous mobile robots. The platform is based on the Xilinx Zynq architecture, a reconfigurable system on chip (SoC) combining a *field programmable gate array* (FPGA) and a dual-core ARM Cortex-A9 processor in a single chip. Standard optical flow implementations have already been realised on the Zynq architecture providing high performance to power ratios combined with high flexibility using HLS (High Level Synthesis) [9]. Within this project, the bio-inspired visual processing on the FPGA is realised using VHDL (Very High Speed Integrated Circuit Hardware Description Language), which enables a high potential for optimization in terms of performance and resource utilization. Additional to the embedded hardware platform a high-speed panoramic camera system allows to emulate the wide field of vision of flying insects. To assess the performance of the embedded processing platform in a real-world scenario the system is used to control visual collision avoidance and navigation behavior on the insect-inspired hexapod walking robot HECTOR (Fig 1A; [10, 11]). Recent work on hexapod robots focusses on different aspects, such as manufactuability [12], different gaits preferable for climbing [13] or bio-inspired visually guided navigation [14]. Due to the mechanical coupling to the ground the application of visually guided navigation on a walking rather than a flying system is challenging as stride-coupled motion of the camera might obfuscate nearness estimation from optic flow [15]. The controller architecture implemented on the embedded hardware module [16] comprises a simple model for insect-inspired visual collision avoidance which has been proposed recently [17]. In contrast to bio-inspired approaches using *binocular vision* [18] or *models of visual motion processing in cortical areas* [19] the model presented here is based on the extraction of nearness information from *optic flow* using so-called *correlation-type elementary motion detectors* (EMDs, [20]). EMDs can be used to model the neuronal mechanisms for estimating optic flow in flying insects [21, 22], avian [23] and other vertebrate species including man (for review, see [24]). The responses of EMDs to pure translational optic flow have been concluded to resemble a representation of the *relative contrast-weighted nearness* to objects in the environment, or, in other words, of the contours of nearby objects [16, 25–28]. The collision avoidance model (a) extracts *nearness information* from optic flow by EMDs, (b) determines a *collision avoidance direction* from the map of nearness estimates and (c) computes a *collision avoidance necessity*, i.e. whether to follow (i.e. potential obstacles are close) or not to follow the collision avoidance direction (i.e. potential obstacles are still rather distant). When coupled with a goal direction, the algorithm has been shown to successfully guide HECTOR to a goal in different simulated cluttered environments while avoiding collisions with obstacles [16]. In this study, the above introduced, bio-inspired algorithms are implemented on a dedicated, resource-efficient hardware and their performance with respect to obstacle avoidance and navigation is optimized in simulation and tested in a minimal real-world scenario with one obstacle in an otherwise visually cluttered environment.

## Embedded hardware platform for bio-inspired visual processing

Insect vision is characterized by a wide field of view, low image resolution and an efficient neural processing of visual information based on the comparatively small computational power of an insect brain. These aspects are mirrored in the presented hardware that was designed to work on mobile platforms like the walking robot HECTOR (Fig 1A). The hardware offers high flexibility and can be deployed as a universal platform for implementing bio-inspired vision algorithms. An integrated high frame rate, high resolution camera allows the adjustment of frame rate and resolution over a wide range of parameters, enabling scalability towards a large number of vision processing approaches. To meet the abilities of the biological example, the hardware platform makes use of raw images from the CMOS-chip and reduces resolution and frame rate to low values efficiently. The preprocessed images are subsequently fed into a bio-inspired model for the processing of visual motion. The hardware platform is mounted in the upper compartment of HECTOR’s front segment (Fig 1B and 1C) and consists of a *processing module*, a *carrier board* providing physical interfaces and power-management functionality, as well as a *high-speed camera system* equipped with a *panoramic fisheye lens*. The main components of the system are described in the following.

### Processing module

**Fig 2:**
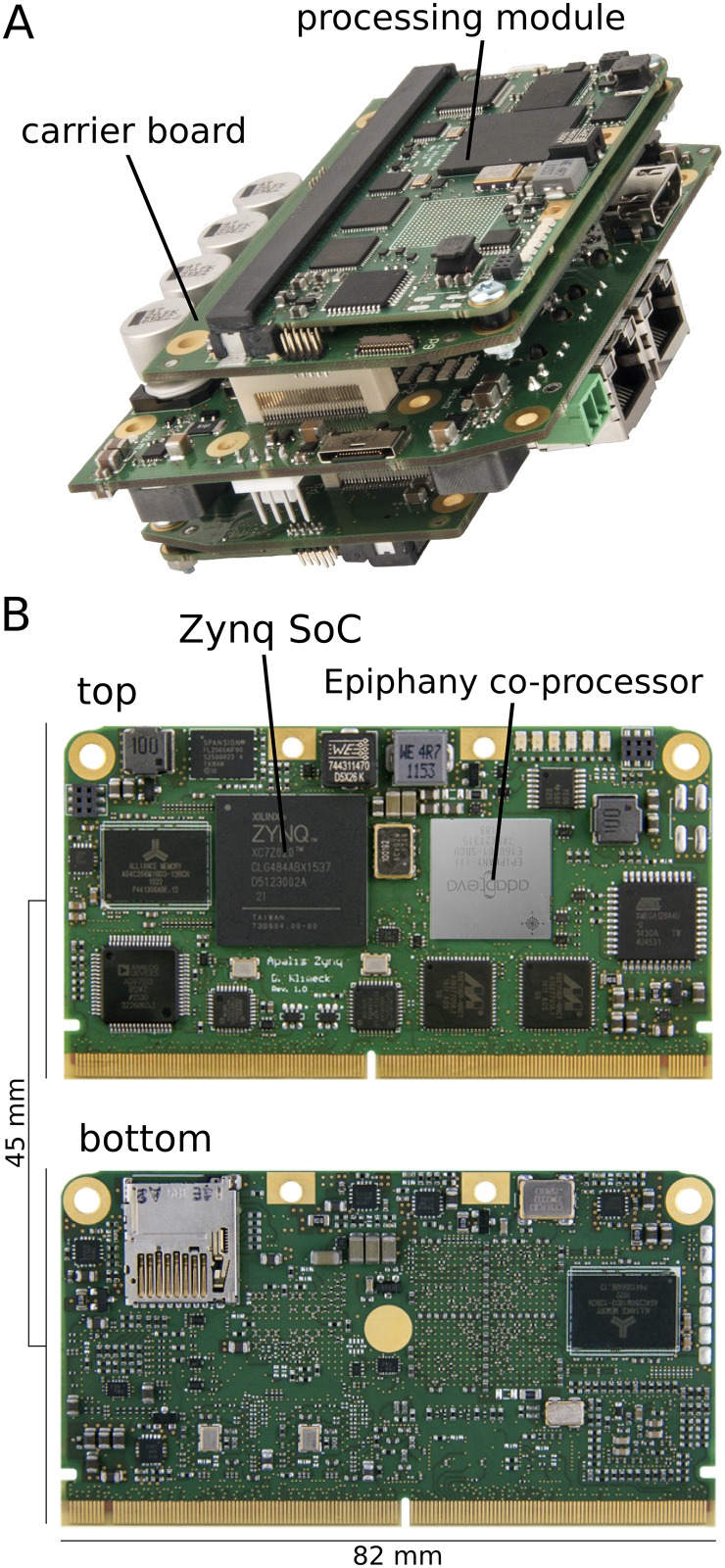
**(A)** The dedicated hardware module for bio-inspired vision processing consists of a *carrier board*—providing physical interfaces and power-management functionality—as well as an *Apalis Zynq processing module*. **(B)** The Apalis Zynq processing module is based on a *Zynq-7000 SoC* and provides a *dual-core ARM Cortex-A9 processor*, an *Artix-7 based programmable FPGA fabric*, as well as an *Epiphany multicore coprocessor*. The processing module allows the highly efficient implementation of bio-inspired algorithms for the processing of visual information provided by the camera system.

The *processing module* (Fig 2B) is based on the Apalis *Computer on Modules* (CoM) standard (Toradex, Horw, LU, Switzerland) and comprises a Zynq XC7Z020-CLG484 SoC (Xilinx, San José, CA, USA) with 1GByte external DDR3 Memory for image processing tasks. The Zynq architecture features a dual-core ARM Cortex-A9 processor running at 667MHz and an Artix-7 based reconfigurable fabric on a single chip. The ARM-CPU and the reconfigurable logic share an AXI communication bus, which allows for high bandwidth and low-latency communication between the on-chip devices. Furthermore, a 16-core Adapteva Epiphany multicore coprocessor (Epiphany E16G301, Adapteva Inc., Lexington, MA, USA) that is directly coupled to the programmable logic of the Zynq-SoC allows for additional processing power. In addition, a dedicated low-power microcontroller (ATxmega128A4U, Atmel Corporation, San José, CA, USA) on the module facilitates power monitoring at runtime.

### Carrier board

For the integration of the processing module in HECTOR, a modular embedded architecture (*carrier board*) has been developed (Fig 2A). The architecture is scalable with respect to its sensor and actuator interfaces and can be easily extended by new processing systems. As a result, the carrier board can be utilized for a wide range of applications. The architecture allows the integration of two Apalis-based CoMs and provides the interfaces for the integration in HECTOR as well as the power supply for the processing modules. To gain further flexibility and compatibility to different robotic platforms, the carrier board is equipped with a broad range of system interfaces (e.g. Gigabit Ethernet, USB, HDMI, CAN, SPI, I^2^C, UART). Moreover, two serial (CSI) and parallel (CPI) camera interfaces allow for a resource- and energy-efficient image data transmission since the data transmission is realized by transferring only the raw image data without additional protocol overhead.

### High-speed panoramic camera system

For the acquisition of images a VITA 2000 camera module (VITA 2000 NOIV1SE2000A-QDC, ON Semiconductor, Phoenix, AZ, USA) is used. The camera module supports frame rates of up to 1730 fps and a resolution of 1920x1080 pixels. Although for the visual collision avoidance and navigation model implemented on HECTOR a frame rate of 20Hz at a resolution of 128x30 pixels proved to be sufficient, a high-speed camera has been chosen, allowing for the implementation of a wide variety of vision processing algorithms, possibly requiring higher frame rates. The VITA 2000 camera is attached to HECTOR’s front segment and is directly coupled to the programmable logic of the Zynq device. The camera’s CMOS sensor delivers images in a raw format (Color Bayer Pattern, 8/10 bit per pixel, linear mode, 60 dB dynamic range) so that no additional protocol overhead is required, resulting in a resource-efficient and frame rate independent image data transmission without storing the incoming image. For internal data transmission, three different AXI-based communication mediums are used inside the FPGA implementation: The AXI-HP bus is used for high data rates, meanwhile the AXI4-Lite bus serves for controlling aspects with lower data rates. Both AXI buses act as an interface between the FPGA and the dual-core CPU inside the Zynq. The third AXI-bus consisting of the AXI4-Stream interfaces the individual bio-inspired IP-Cores to transmit the visual data in a resource-efficient way. The *Camera Receiver* core inside the FPGA (see Fig 4) allows access to the image data of the camera via an AXI4-Stream bus [29] and furthermore enables the configuration of the camera via the processing system by means of an AXI4-Lite bus. Additionally, the camera is equipped with a non-linear fisheye lens (Lensagon BF2M2020S23, Lensation GmbH, Karlsruhe, Germany) with a circular field of vision (FOV) of 195° to emulate the wide field of view of flying insects [30]. The weight of the dedicated embedded hardware module (Fig 2A) merely amounts to 178 g with a size of 10 cm x 4.2 cm x 8 cm. The related camera module containing the fisheye lens (Fig 1C) contributes additional 39 g and a size of 3 cm x 4 cm x 4 cm to the overall visual processing system that results to a total weight of 252 g, including the high-speed camera cable (Fig 1B).

## Vision-based direction controller

**Fig 3:**
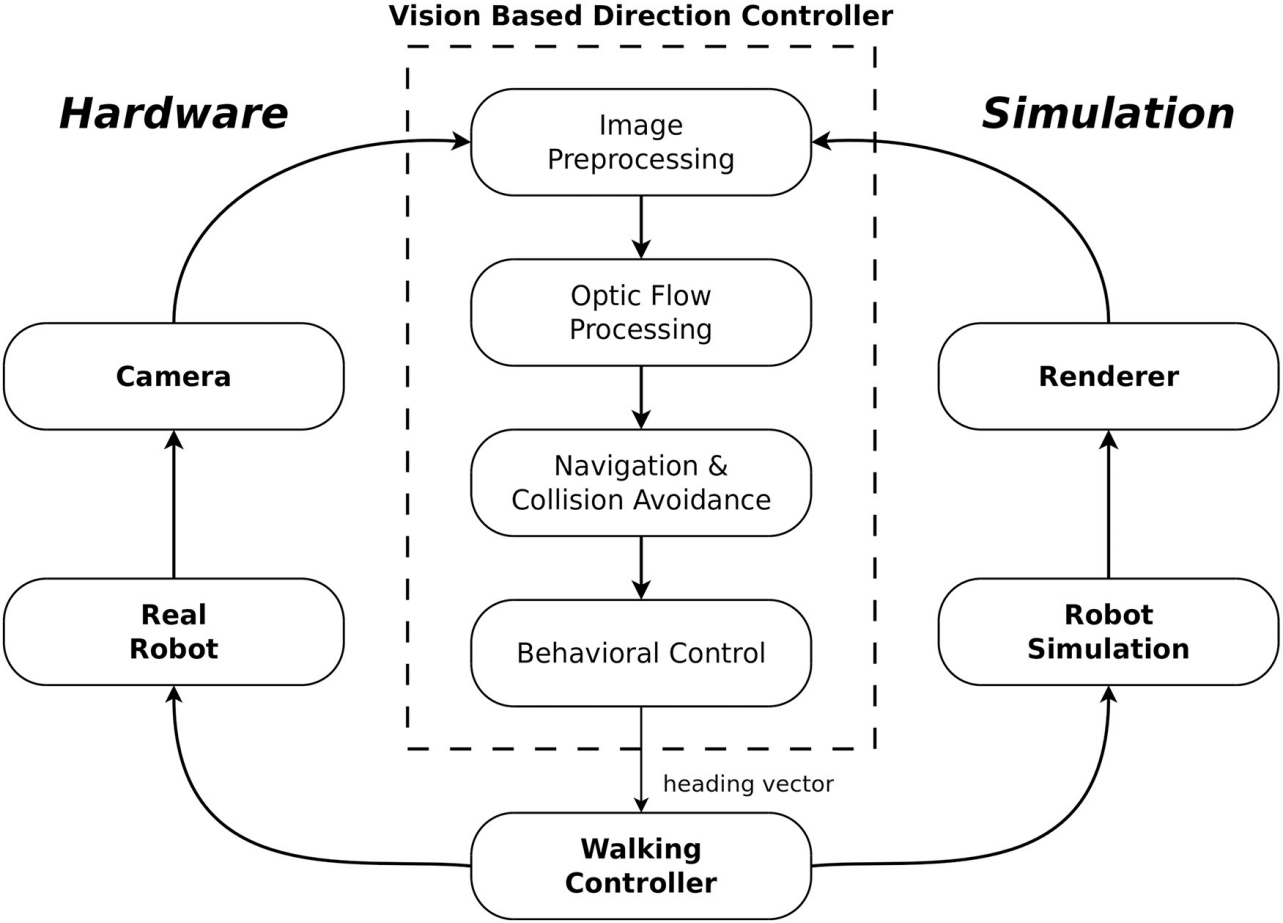
The controller framework used for implementing the visual collision avoidance model in simulation and on the embedded hardware module [16]. The dashed box (*Vision-Based Direction Controller*) indicates the algorithm used for controlling HECTOR’s behavior based on nearness estimation from optic flow.

To enable HECTOR to navigate in cluttered environments a vision-based direction controller framework (Fig 3; [16]) was implemented on the embedded hardware platform. The controller is based on a recently proposed model for insect-inspired visual collision avoidance [17]. In a previous simulation study it could be shown that the controller framework enables HECTOR to successfully navigate to goal positions in different cluttered environments while avoiding collisions solely based on the extraction of nearness information from optic flow. The controller processes the sequences of images obtained from the panoramic camera system based on four consecutive steps (Fig 3; see [16]):

1. *preprocessing of images*, in order to emulate the characteristics of the visual input of flying insects,
2. estimation of a relative nearness map by *processing of optic flow* via EMDs,
3. computation of a *collision avoidance direction* based on the relative nearness of objects and a goal direction, and
4. *controlling the walking direction* of the robot.

The hardware-based realization of the pixel-based processing that consists of the processing steps a)—b) is realized inside the FPGA of the Zynq SoC, while the feature extraction described in c) is implemented on the dual-core CPU of the Zynq. This hardware-software-partitioning allows a highly resource-efficient parallelized implementation of the bio-inspired visual processing. The final processing step d) is outsourced to the main processing system placed in the middle segment of HECTOR. The details of all processing step are described in the subsequent sections. The respective processing cores inside the FPGA were implemented using the hardware description language VHDL.

### Preprocessing of images

Flying insects, such as flies or bees, are able to perform complex visually-guided behaviors with a minimum of neuronal computing power. Hence, in the vision-based direction controller used to control collision avoidance and navigation behavior on HECTOR [16] aspects of the early visual stages of the visual system of flies are adopted. Due to the large panoramic visual field and the low spatial resolution of the visual system of flies [30] the computational requirements of processing optic flow can be reduced, since optic flow induced on the retina by self-motion or motion in the environment can be processed with a relatively small number of computational units. Hence, the preprocessing of camera images is based on a) *remapping and scaling* of the visual input to emulate the relatively low spatial resolution of the fly’s compound eye, as well as, b) *brightness adaptation* to varying light intensities, such as performed by photoreceptors and peripheral visual interneurons [31, 32], which allows flies to navigate their environment even under dynamic brightness conditions [26].

#### Remapping and scaling

The non-linear fisheye lens attached to the camera possesses distortion characteristics, that visually enlarges objects along the optical axis of the lens. Objects near the periphery occupy a smaller area of the image. Thus, objects in the vicinity of the optical axis are transmitted with much greater detail than objects in the peripheral viewing region, which obfuscates nearness estimation from optic flow. Hence, images obtained from the fisheye lens are remapped to a rectilinear representation [33] according to

$$\begin{array}{c} R_{u}=f\cdot \tan(2\cdot \arcsin\frac{R_{d}}{2f}), \end{array}$$ (1)

where *R*_*u*_ represents the undistorted radial pixel position after remapping, *R*_*d*_ the radial pixel position in the image obtained from the camera and *f* the focal length of the fisheye lens.

The compound eye of insects consists of a two-dimensional array of hexagonally aligned ommatidia comprising the retina. Each ommatidium consists of a lens that forms an image pixel of the surrounding world onto the top of a rhabdom, a light-guiding structure of photopigment-containing membrane that is formed by a small number of photoreceptor cells [34]. The image points sensed by the hexagonally arranged ommatidia form the actual images that serve as the input into the visual system. Although blowflies possess color vision, evidence suggests that the pathways involved in motion detection are monochromatic [35]. Therefore, only the green color channel of the camera images is used. To mimic the relatively low spatial resolution of the compound eyes of flies [30], each camera image is scaled down to a rectangular grid of photoreceptors with an interommatidial angle of 1.5°, resulting in an array of 128x30 pixels (i.e. luminance values) covering a field of view of 192° horizontally and 45°vertically [16].

**Fig 4:**
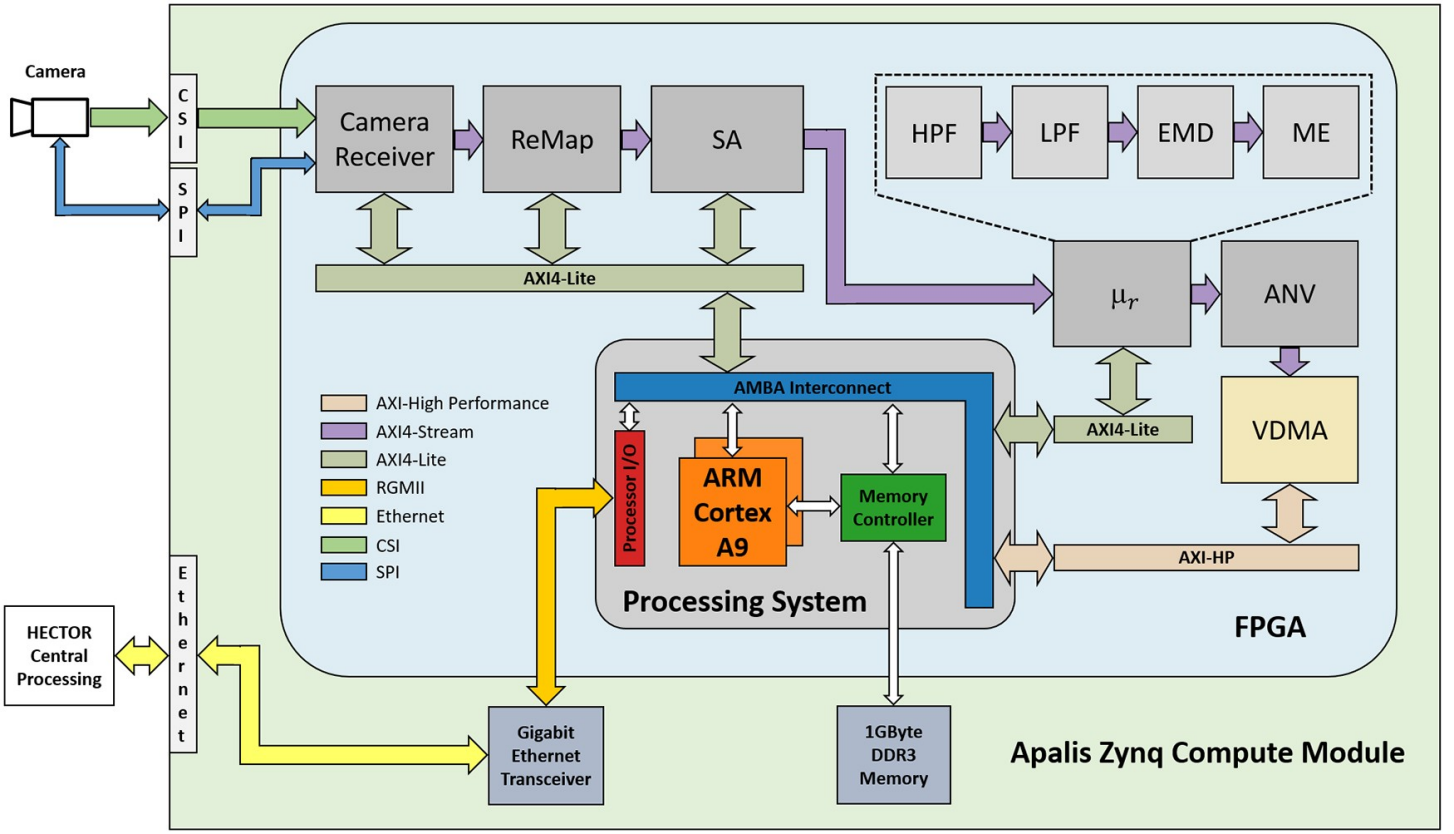
The bio-inspired vision processing framework implemented in HECTOR is realized by the novel Apalis Zynq CoM (green box). The processing of the image data is implemented within the Xilinx Zynq SoC (blue box) consisting of a programmable logic (FPGA) and a dual-core CPU (ARM Cortex-A9). The computationally expensive steps of the vision-based direction controller are implemented via FPGA-based IP-Cores while the remaining processing steps are implemented in software on the CPU. (Explanation of abbreviations: CSI: Camera Serial Interface; SPI: Serial Periphal Interface; ReMap: Remapping and downscaling IP-Core; SA: Sensivity Adaption IP-Core; *μ*_*r*_: Contrast-weighted relative nearness Map IP-Core; HPF: High-Pass Filter IP-Core; LPF: Low-Pass Filter IP-Core; EMD: Elementary Motion Detector IP-Core; ME: Motion Energy IP-Core; ANV: Average Nearness Vector IP-Core; VDMA: Video Direct Memory Access IP-Core; AXI-Lite: Advanced eXtensible light-weight Interface Bus; AXI-HP: Advanced eXtensible high-performance Interface Bus).

The remapping and pixel-wise downscaling of the camera images is implemented in the *ReMap* core on the FPGA (see Fig 4). The stream-based processing of the image data is realized using a *look-up table* (LUT), thus enabling a frame rate independent (low latency) processing. The Matlab toolbox *OCamCalib* [36] was used for the generation of the mapping table and the corresponding coefficient files that are stored in the internal memory of the FPGA. This stream-based approach based on mapping tables significantly reduces the required storage by a factor of 28 inside the FPGA compared to the direct computation of the image remapping. Since the remapping is directly performed on the incoming video stream, no memory is required for data buffering.

#### Brightness adaptation

Facing a vast dynamic range of light intensities, visual systems have to adjust their operating range according to the prevailing light conditions. The *sensitivity adaptation* (*SA*) core (see Fig 4) implemented on the FPGA adopts the mechanism of brightness adaptation of the photoreceptors and the early processing stages of the visual system of insects. Since photoreceptors have to cope with a wide range of light intensities, while their operating range is limited, they adjust their sensitivity dynamically to the current brightness level [31, 32]. In contrast to the insect’s visual system the brightness adaptation is implemented *globally* within the *SA* core using *gamma correction*, which maps an input image *g* to a brightness-adapted image *g*′:

$$\begin{array}{c} g^{\prime }\left(g\right)=w_{max}\cdot {\left(\frac{g-w_{min}}{w_{max}-w_{min}}\right)}^{\gamma_{c}}+w_{min}. \end{array}$$ (2)

The maximum value of the prevailing light intensity is indicated by *w*_*max*_, the minimum by *w*_*min*_, respectively. The gamma value *γ*_*c*_ depends on the mean value of the input image *g*_*mean*_ as well as the mean value of the dynamic light intensity range and can be adjusted dynamically according to

$$\begin{array}{c} \gamma_{c}=\frac{g_{mean}}{w_{mean}}. \end{array}$$ (3)

Gamma correction is implemented within the FPGA via a gamma correction IP-Core provided by Xilinx. The mean luminance value of the input image *g*_*mean*_ is calculated for each frame by the *ReMap* core and then read by the *SA* core via the AXI4-Lite bus (Fig 4). Based on the mean luminance value, the computation of the gamma value *γ*_*c*_ and the gamma correction are performed by the processing system. Finally, the updated mapping function is written to the *SA* core via an AXI4-Lite bus. The range of ambient light conditions HECTOR is able to compensate for equals 56 dB by using 8 bit per pixel resolution and 60 dB for 10 bit resolution. Compared to 8 bit resolution, the precision of the measured light intensity increases by a factor of 4 when 10 bit resolution is applied. Therefore, the use of 10 bit per pixel resolution is advantageous for the brightness adaption, especially in low-contrast environments. In natural environments with high contrast the 8 bit variant is adequate with respect to brightness adaptation and beneficial in terms of resource efficiency.

### Processing of optic flow

In flying insects as well as in vertebrate species, optic flow is estimated by a mechanism that can be modelled by correlation-type elementary motion detectors (EMDs; [20]). In the vision-based direction controller [16], optic flow estimation is based on two retinotopic arrays of either horizontally or vertically aligned EMDs. Individual EMDs are implemented on the FPGA within the *EMD* core (Fig 4) by a multiplication of the delayed signal of a receptive input unit with the undelayed signal of a neighboring unit. Only interactions between direct neighbors are taken into account for both horizontally and vertically aligned EMDs. The luminance values from the photoreceptors are filtered with a first-order temporal high-pass filter (*HPF* core; *τ*_*hp*_ = 20 ms) to remove the mean from the overall luminance of the input. The filtered outputs are subsequently fed into the horizontally and vertically aligned EMD arrays. The delay operator in each halfdetector is modelled by a temporal first-order low-pass filter (*LPF* core; *τ*_*hp*_ = 35 ms). The time constants *τ*_*hp*_ and *τ*_*lp*_ are adjustable by the processing system via the AXI4-Lite bus during runtime. Each EMD consists of two mirror-symmetric subunits with opposite preferred directions. Their outputs are subtracted from each other. For each retinotopic unit the motion energy *μ*_*r*_(*x*, *y*) [25] is computed within the *motion energy* (*ME*) core (see Fig 4) by taking the length of the motion vector given by the combination of the responses of a pair of the horizontal *h*_*EMD*_ and the vertical *v*_*EMD*_ at a given location (*x*, *y*) of the visual field according to

$$\begin{array}{c} \mu_{r(x,y)}=\sqrt{v_{\text{EMD}}^{2}\left(x,y\right)+h_{\text{EMD}}^{2}\left(x,y\right)}. \end{array}$$ (4)

The array of the absolute values of these local motion vectors *μ*_*r*_ due to translatory self-motion resembles a map of *contrast-weighted relative nearness* to objects in the environment, providing information on the contours of nearby objects [25, 26].

### Computation of the collision avoidance direction

Once the relative nearness map *μ*_*r*_ has been computed, collision avoidance is achieved by moving away from the maximum nearness value (e.g. objects that are close). However, the contrast-weighted nearness map also depends on the textural properties of the environment. To reduce the texture dependence, the nearness map is averaged along the elevation *ϵ* in the *ANV* (average nearness vector) core on the FPGA (see Fig 4), giving the *average nearness* for a given azimuth *ϕ* [17]. Each of these averaged nearness values can be represented by a vector in polar coordinates. The norm of this vector is the averaged nearness and its angle corresponds to the azimuth. The averaged nearness values are transmitted to the ARM-processor via an AXI-High Performance port into an additional *video direct memory access* core (*VDMA*). The sum of these vectors points towards the average direction of close objects. This vector is denoted *center-of-mass-average-nearness-vector* (*COMANV*, [17]) and is computed by the ARM Cortex-A9 processor based on the output data of the *ANV* core:

$$\begin{array}{c} COMANV=\sum ((\begin{array}{c} \cos(\phi )\\ \sin(\phi ) \end{array})\frac{1}{n}\sum \mu_{r}(\epsilon ,\phi )). \end{array}$$ (5)

The parameter *n* describes the number of elements in the azimuth. The inverse of the *COMANV* vector, scaled to the horizontal field of view *θ* of the photoreceptor array, points away from the closest object and, thus, can be used as the direction of the robot to avoid collisions (*collision avoidance direction*, *CAD*_*fov*_, [16, 17]):

$$\begin{array}{c} CAD_{fov}=\frac{-\arctan\left(COMANV_{y},COMANV_{x}\right)}{\frac{2\pi }{\theta }}. \end{array}$$ (6)

The length of the *COMANV* vector increases with nearness and apparent size of objects. Its length is a measure of the collision avoidance necessity *CAN*:

$$\begin{array}{c} CAN=∥COMANV∥. \end{array}$$ (7)

The *CAD*_*fov*_ as well as the *CAN* value are calculated by the ARM-Processor and then transmitted to HECTOR’s central processing system via Ethernet.

### Controlling the walking direction

In the vision-based direction controller the *CAN*-measure and the collision avoidance direction *CAD*_*fov*_ obtained from the embedded processing platform is used to control the heading direction *γ* of the robot to compromise between *avoiding collisions* and *following the direction to a goal* [16]:

$$\begin{array}{c} \gamma =W\left(CAN\right)\cdot CAD_{fov}+\left(1-W\left(CAN\right)\right)\cdot \alpha . \end{array}$$ (8)

**Fig 5:**
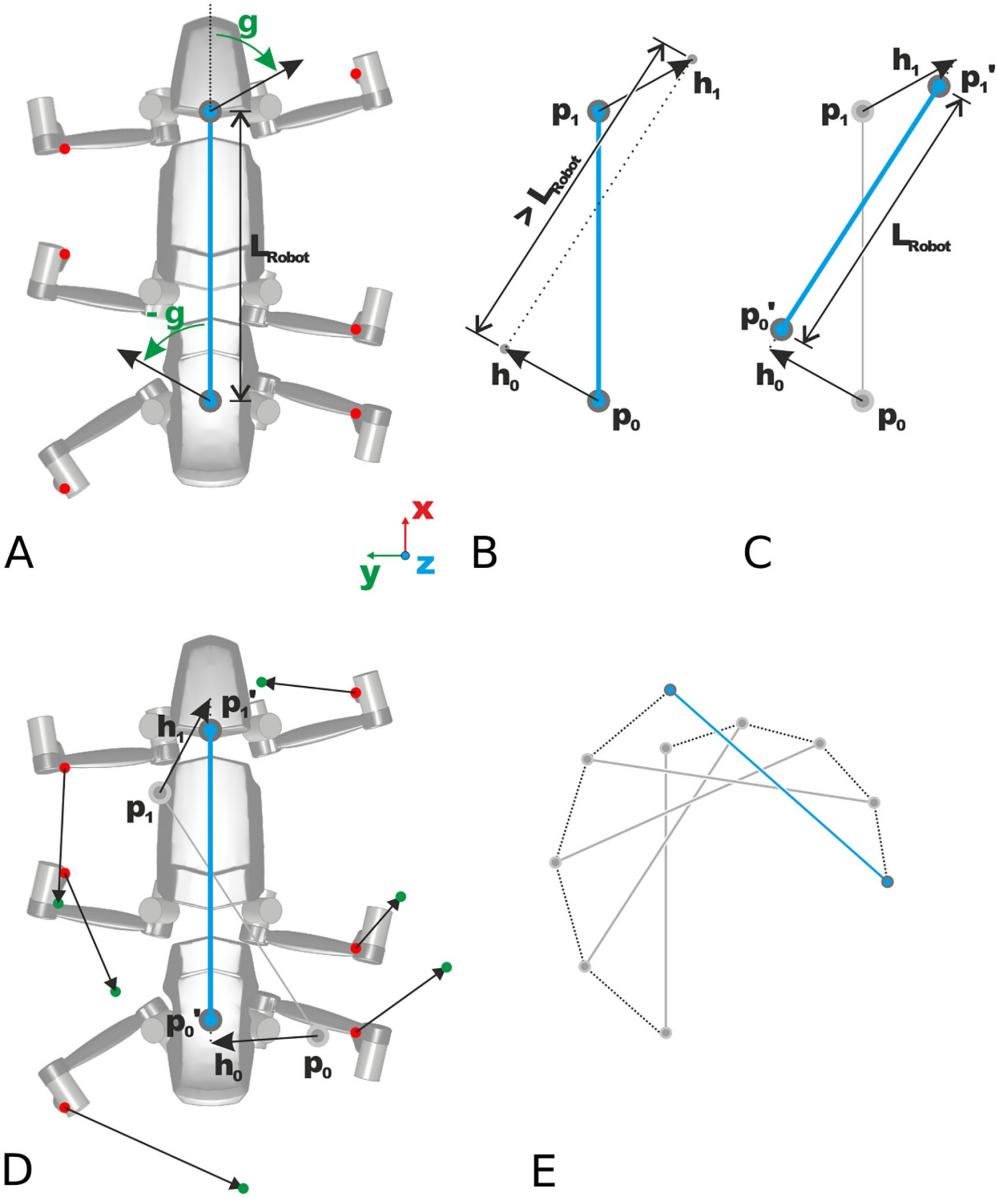
Body model for generation of stance trajectories in omnidirectional walking. (A) Body model (top view) with pull points between front leg coxae and hind leg coxae which define the end points of the longitudinal body axis (blue). Control vectors with angle *γ* w.r.t. the longitudinal body axis are constructed at the pull points. These vectors define the displacement of the pull points (B) and lead to a rotation and shift of the longitudinal axis which is normalized to the robot length afterwards (C). (D) Rotation and shift of longitudinal body axis lead to a displacement of the foot points of all legs with ground contact (displacements calculated with body model). These displacements are used in the single leg controllers to move the robot on a desired trajectory. (E) Exemplary movement of the body axis with constant heading direction *γ*.

*W* is a sigmoid weighting function based on the *CAN*:

$$\begin{array}{c} W\left(CAN\right)=\frac{1}{1+{\left(\frac{CAN}{n_{0}}\right)}^{-g}}, \end{array}$$ (9)

and driven by a gain *g* and a threshold *n*_0_ [17]. To reduce the influence of abrupt directional changes induced by the estimation of the *CAD* from optic flow the heading direction *γ* is filtered with a temporal first order low-pass filter (*τ*_*lp*_ = 20 ms). The temporally filtered heading direction *γ*_*lp*_ is subsequently used to directly control the walking direction of HECTOR. The walking controller is based on the WALKNET concept of Cruse [37, 38]. In this concept, the legs are separate agents that independently carry out swing and stance movements. In order to obtain a coordinated walking pattern, neighboring legs exchange information about their current state and evaluate it with the so-called coordination rules [37]. The information exchanged between the legs includes, for example, whether a leg is in its swing or stance phase, the distance of the leg from its lifting point (swing to stance transition) or the strength of its momentary mechanical load. In addition to the coordination to achieve a cyclical, adaptive walking pattern, the locomotion itself must also be given a direction. In concrete terms, this means that all legs in their stance phase must choose the direction of their stance movement in such a way that the central robot body moves in the desired direction. This goal is achieved with the help of an internal body model, which represents the kinematic structure of the robot (see Fig 5A).

In order to specify a walking direction, pull points (*p*_0_, *p*_1_, blue) are defined centrally between the attachment points of the front and hind legs. The connection line between the two pull points (*p*_0_, *p*_1_) represents the longitudinal axis of the robot’s body. At both pull points, a control vector with the angle *γ* (heading direction) relative to the body axis is constructed. The length of the control vector correlates with the desired movement speed of the robot. As shown in Fig 5B and 5C, the pull points (*p*_0_, *p*_1_) are shifted according to the control vector ($p_{0}^{\prime },p_{1}^{\prime }$) and their distance is then normalized to the robot length. Since the positions of all foot points of legs with ground contact must be maintained, the body model can be used to determine the corresponding displacement vectors for the individual legs and to generate individual stance movements (Fig 5D). Thus, a path trajectory for the longitudinal axis of the robot body is obtained, as exemplary shown in Fig 5E. The explicit version of the body model used here can also be replaced by a body model based on neural networks [39].

For the control of visual collision avoidance of HECTOR, the temporally low-pass filtered heading direction *γ*_*lp*_ is fed into the walking controller. However, due to the camera’s restricted horizontal field of view, no information on the nearness of objects outside of the field of view can be obtained. Nevertheless, as the camera is pointing forward along the direction of walking during translation, information on the nearness of objects sidewards or behind the robot is not essential. In situations where the goal direction *α* does not reside within the field of view, the *CAN* is set to zero, effectively inducing a turn of the robot until the goal direction lies within the camera’s field of view [16].

## Visual collision avoidance in a real-world scenario

**Fig 6:**
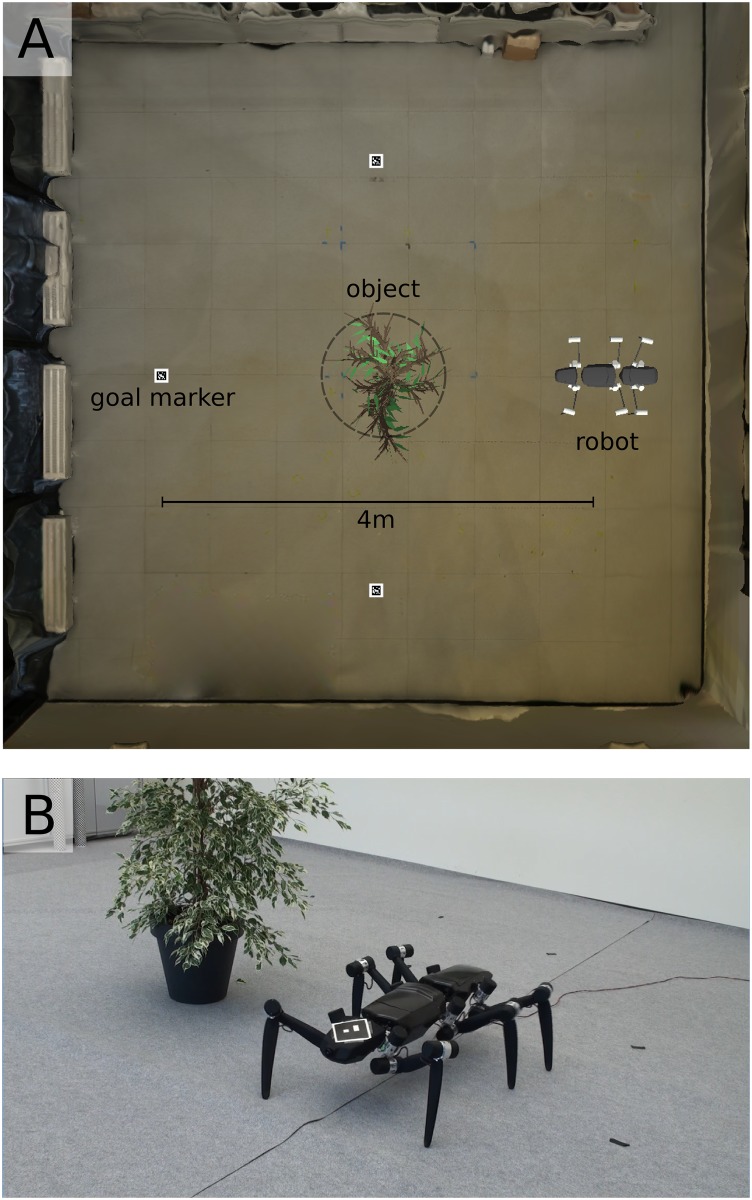
**(A)** Software simulation of the experimental setup (top view). A 3D reconstruction of the Teleworkbench was used to optimize the threshold *n*_0_ and gain *g* parameters of the vision-based direction controller in simulation. In the experimental setup visual markers (*goal markers*) were placed to indicate four different goal positions. The *robot* was placed 4 m apart and opposite of the goal position facing an *object* (i.e. a bush) located in the center of the arena. An experimental trial was successful, when the robot reached the goal position without it’s front segment crossing a radius of *r* = 0.5 m around the object (*dashed circle*). **(B)** Experimental trial in the real world. After parameter optimization the visual collision avoidance task was performed on the physical robot. A visual marker was placed on top of the robot’s front segment to obtain the relative direction to the goal *α* [Eq (8)].

The performance of the collision avoidance and vision-based direction control on the embedded processing platform was assessed in a confined experimental navigation task (see Fig 6). The task required the robot to reach different goal positions in an experimental arena while avoiding collisions with an object (i.e. a bush) placed in the center of the arena. In a first step, the parameters threshold *n*_0_ and gain *g* of the vision-based direction controller were optimized in a software simulation of the experimental paradigm (Fig 6A). Based on the optimized values, the performance of the vision-based direction and collision-avoidance controller embedded in the physical hardware was assessed in a real-world experiment in the arena (Fig 6B).

### Experimental paradigm

To evaluate the performance of visual collision avoidance and navigation on the embedded vision processing platform experiments were performed in the *Teleworkbench* (TWB; see Fig 6; [40]) located at CITEC, Bielefeld University. The TWB offers a standardized environment for the evaluation of autonomous robotic agents and comprises a main experimental arena of 7 m x 7 m. The arena can be partitioned into four sub-fields. Four 1-megapixel cameras are mounted above the experimental arena and are assigned to one sub-field each. Each camera is connected to a video server that processes the video data to provide the 3D-position and orientation of visual markers (see Fig 6) located within the arena. In the experimental paradigm the visual markers were used to obtain the global position and orientation of the robot’s front segment as well as to mark four goal positions within the arena (see Fig 6A). The robot was placed at either one of four starting positions in front of an object (i.e. a bush), located in the center of the arena, facing a goal position behind the object (see Fig 6A). The distance between starting position and goal was set to 4 m. An experimental trial was assumed to be successful, if the robot reached the goal position without the robot’s front segment crossing a radius of 0.5 m around the object (*dashed circle* in Fig 6A).

### Parameter optimization in simulation

In a first step, the initial parameters of the vision-based direction controller consisting of the threshold *n*_0_ and gain *g* were optimized in a software simulation of the experimental paradigm. Hence, in the first part of the experiment, a three-dimensional model of the TWB (see Fig 6A) was used to render images of the camera attached to the robot’s front segment and emulate the processing steps of the vision-based direction controller as implemented on the embedded hardware module [16]. The robot’s orientation and position as well as the location of the goal were obtained from a software module emulating the positional tracking of the TWB.

**Fig 7:**
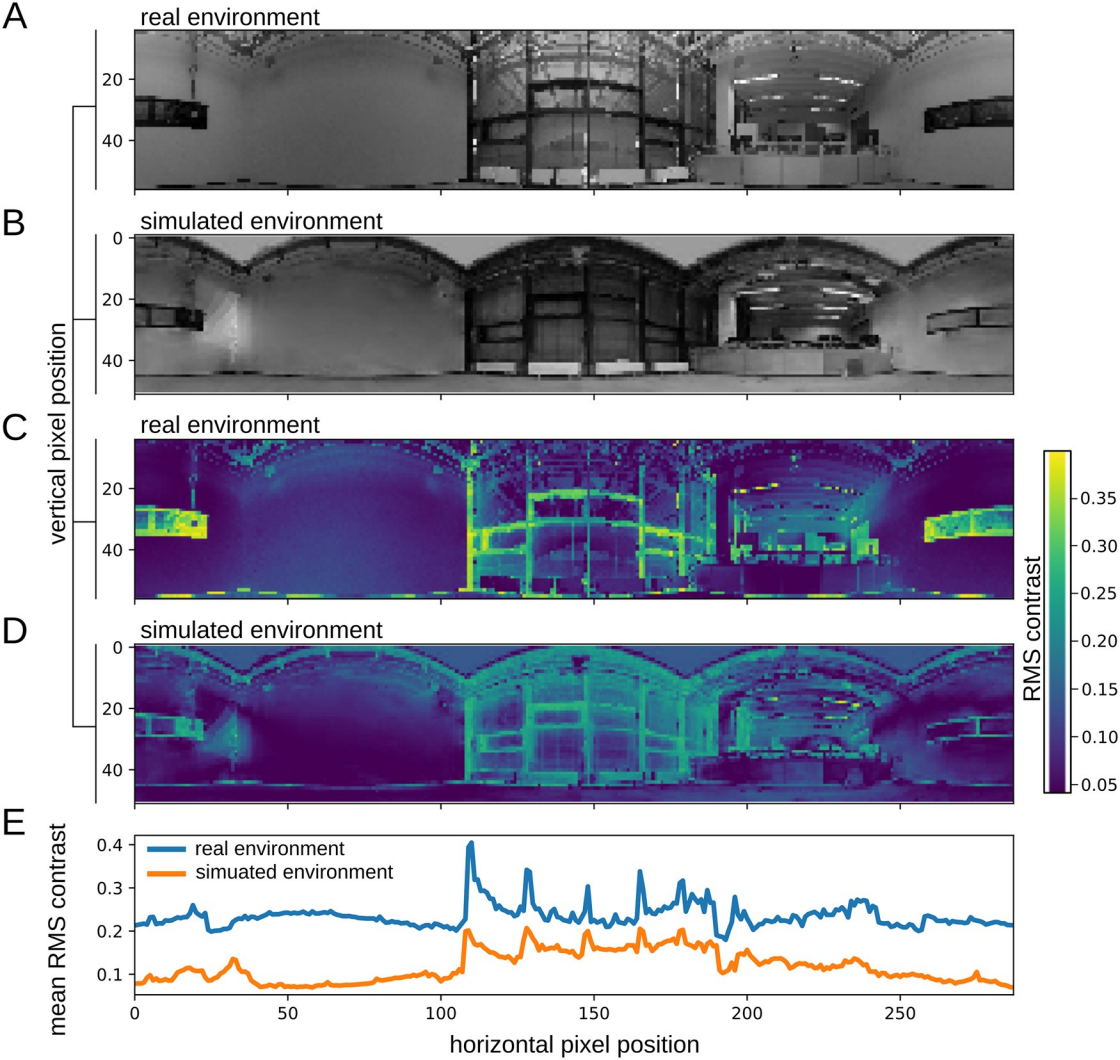
360° panoramic images of (A) the *real environment* and (B) the *simulated environment* reconstructed using photogrammetry. Both images represent the orthographic reprojections using a cylindrical lens positioned at the center of the experimental arena (*x*, *y* = [0.0, 0.0]). (C) and (D) depict the color-coded local root-mean-square (RMS) contrast. To compare the local contrast distributions along the horizontal axis for the real and the simulated environments, the vertical mean RMS contrast was computed (E).

To obtain the three-dimensional model of the experimental arena a set of 120 digital photographs taken from different positions and viewing angles were used to reconstruct the TWB using photogrammetry software (Autodesk ReMake, Autodesk Inc., San Rafael, CA, USA). The extraction of nearness information from optic flow via EMDs, such as performed by the vision based direction controller, strongly depends upon the local contrast of the input images [25]. Hence, the local contrast of the 360° panoramic images of the *simulated* and of the *physical* environment (see Fig 7A–7D) was computed as the *root mean square* (RMS) *contrast* between each pixel of the image down-sampled to ommatidial resolution, and its four direct orthogonal neighbors. The RMS contrast was calculated by taking the standard deviation of the brightness *I*(*x*, *y*) of all pixels (*x*, *y*) of the local region divided by the mean brightness *I* of the same region [41].

Although the mean of the average local contrast distributions along vertical extent of the images differ (see Fig 7E), prominent features are locally discernible in both environments. Hence, a similar performance of the vision-based direction controller in the simulation as well as in the physical environment using the same parametrization of the controller was expected.

**Fig 8:**
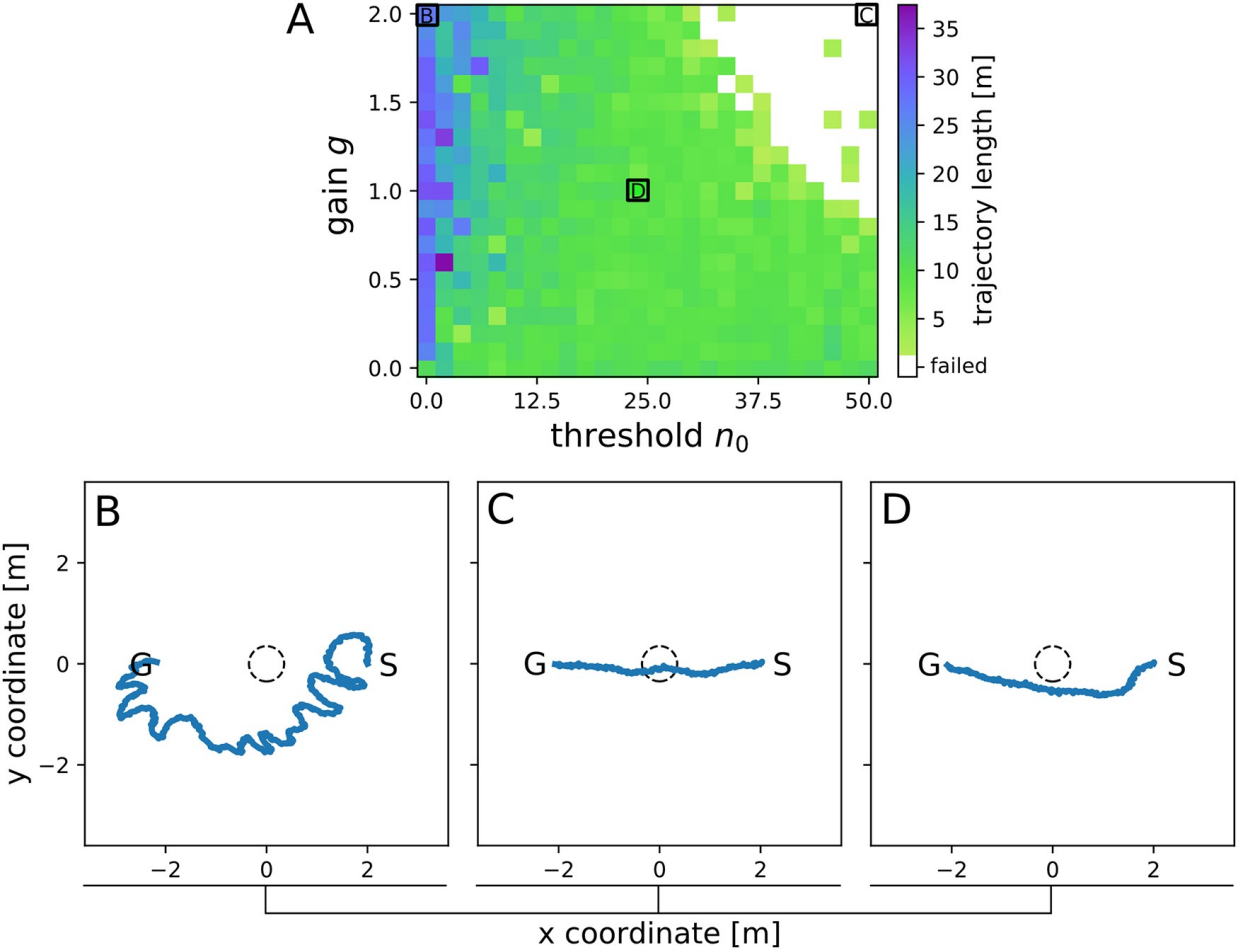
(A) Length of simulated trajectories *(color-coded)* in the simulated environment (see Fig 6A) for different combinations of the weighting function parameters gain *g* = [0.0, 0.1, …, 2.0] and threshold *n*_0_ = [0.0, 2.0, …, 50.0] [see Eq (9)]. The size of the simulated environment is 7 m x 7 m (length x width). When the trajectory crossed a circle of a radius of 0.5m around the center of the object (dashed line in B-D) a collision was assumed (white areas). B-D) Simulated trajectories (n = 10) in the reconstructed environment. Starting positions are given as S and goal positions as G. Weighting function parameters were set to (B) *g* = 2.0 and *n*_0_ = 0.0, (C) *g* = 2.0 and *n*_0_ = 50.0 and (D) *g* = 1.0 and *n*_0_ = 24.0.

For each of the possible parameter combinations of the gain *g* = [0.0, 0.1, …, 2.0] and threshold *n*_0_ = [0.0, 2.0, …, 50.0] the mean trajectory length (n = 3 trials) for reaching each of the four goal positions without colliding with the object was taken as a benchmark of the performance of the vision-based direction controller in simulation (see Fig 8A). When the threshold *n*_0_ is set to low values, the computation of the heading direction *γ* [see Eq (8)] mainly depends on the collision avoidance direction *CAD*_*fov*_, whereas the goal direction *α* is only taken into account to a small extent. Hence, the robot will more likely avoid collisions than navigate to the goal (G). Further, a steeper slope of the sigmoid weighting function *W*, set by the gain *g*, leads to higher temporal fluctuations of the heading direction *γ*. As a consequence, when setting the threshold to *n*_0_ = 0.0 and the gain to *g* = 2.0, the resulting trajectories were relatively long (Fig 8A) and showed erratic movement patterns as can be seen in the example trajectory depicted in Fig 8B. In contrast, when setting the threshold *n*_0_ to high values, the computation of the heading vector *γ* mainly takes the goal direction *α* into account, whereas the influence of the collision avoidance direction (*CAD*_*fov*_) is reduced. As a consequence, the robot will more likely follow the direction to the goal without avoiding obstacles [16]. Therefore, when setting the threshold to *n*_0_ = 50.0 and the gain to *g* = 2.0, the robot directly approached the goal position, consequently, colliding with the object (Fig 8A and 8C). Fig 8D shows an example of a trajectory for a combination of the parameters gain and threshold which resulted in short trajectory lengths without collisions (*n*_0_ = 24.0, *g* = 1.0). Here, the robot almost directly approached the goal, while effectively avoiding the object. This combination of the threshold and gain parameters was implemented on the physical robot to test the performance of the embedded hardware module in subsequent experiments in the TWB.

### Collision avoidance in the real world

**Fig 9:**
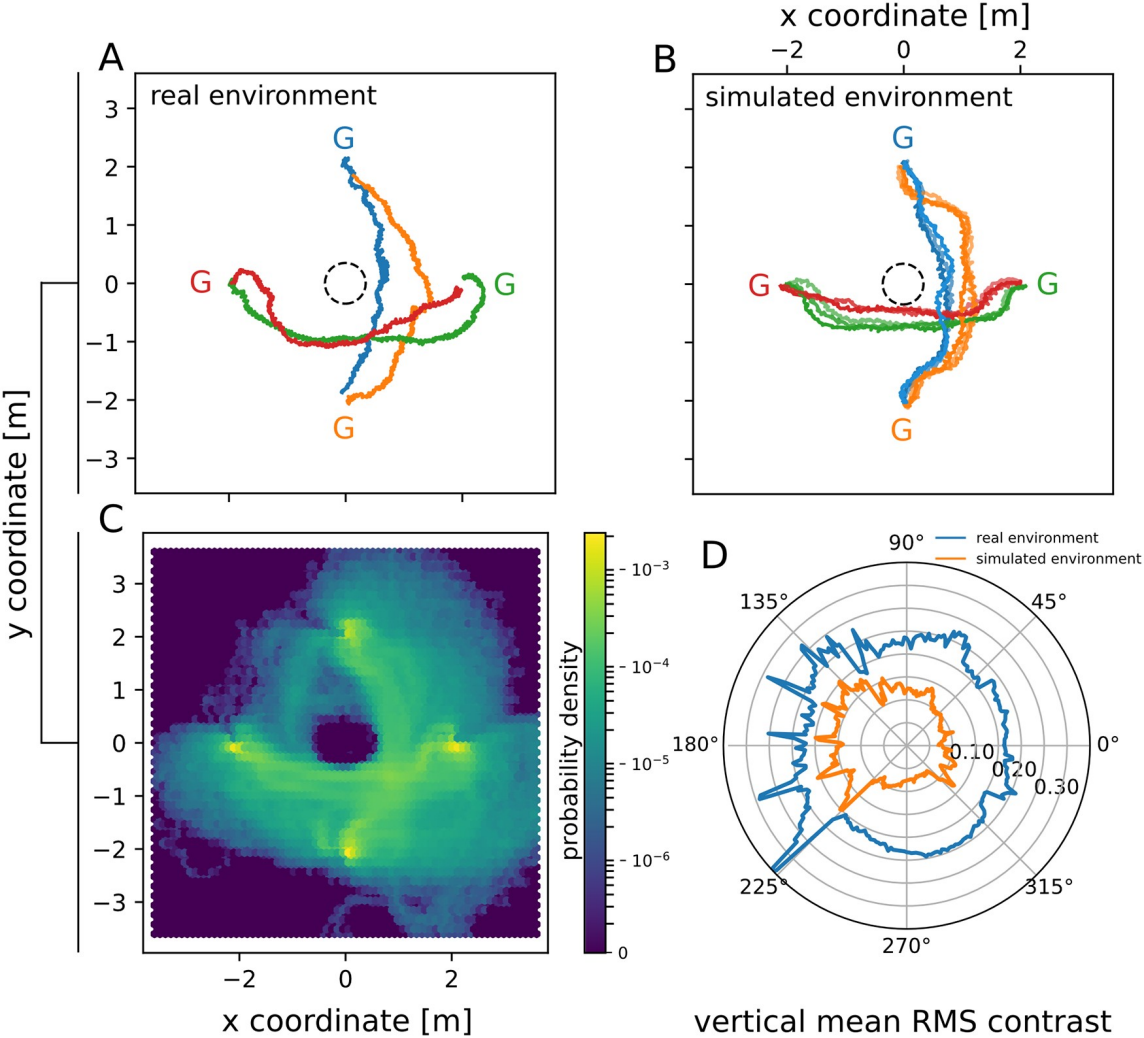
**(A)** Trajectories obtained in the real-world scenario for different goal positions (color-coded; G). The dotted circle (*r* = 0.5 m) indicates the object (bush) located in the center of the arena. The distance between the starting position and the goal position was 4 m. **(B)** Trajectories obtained in simulation for different goal positions (color-coded; G). For each goal position n = 3 trials were performed. **(C)** Spatial probability density distribution for all trajectories obtained from simulation. Different combinations of the weighting function parameters [see Eq (9)] threshold *n*_0_ = [0.0, 2.0, …, 50.0] and gain *g* = [0.0, 0.1, …, 2.0] were used. For each parameter combination n = 3 trials were performed. **(D)** Vertical mean RMS contrast plotted in polar coordinates as also depicted in Fig 7E.

After optimization of the parameters in simulation, the performance of the vision-based direction controller was evaluated on the physical robot. In order to obtain the relative direction to the goal *α* a visual marker was placed at the robot’s front segment as well as at the goal position in the respective experimental trial (Fig 6B). For each goal position (*n* = 1) trial of the visual collision avoidance task was performed. In each trial the robot was able to successfully reach the goal position without colliding with the obstacle (i.e. crossing a radius of *r* = 0.5 m around the object) as can be seen in Fig 9A. When comparing the trajectories obtained from the physical robot with the trajectories obtained from simulation with the same threshold *n*_0_ and gain *g* parameters (Fig 9B), a similar performance can be observed. However, in the real-world scenario the robot kept a higher distance to the object while avoiding collisions. This behavior can most likely be attributed to imperfections in the simulation of the robot’s walking aparatus resulting in different optic flow patterns obtained by the camera, as well as in the virtual reconstruction of the experimental arena. A higher mean amplitude of local contrast distributions, as measured in the real environment, leads to a higher influence of the collision avoidance direction *CAD*_*fov*_ [Eq (6)] when computing the heading direction *γ* [Eq (8)]. Hence, the physical robot moves away from the object more strongly as compared to the collision avoidance behavior observed in simulation.

Further, it is noteworthy that the trajectories show a tendency of the robot to navigate around the object along certain boundaries of the experimental arena both in the real scenario and in simulation (Fig 9A and 9B; arena boundaries are represented by the edges of the axes). When comparing the local probability density distribution of the trajectories for all simulation trials (Fig 9C) with the mean vertical local contrast distribution within the experimental arena (Fig 9D) it can be seen, that the vision-based direction controller preferably chooses routes along areas of low spatial frequency and contrast. A characteristic property of EMDs is that the output does not exclusively depend on velocity, but also on the pattern properties of a moving stimulus, such as its contrast and spatial frequency content [42, 43]. Furthermore, the vision based direction controller is most sensitive to vertical texture patterns, due to the averaging of EMD outputs along the elevation *ϵ* [see Eq (5)]. Hence, nearness information can not be extracted unambiguously from EMD responses, as visual motion consisting of vertical low contrast and spatial frequency patterns induces a lower EMD response amplitude as compared to vertical high contrast and spatial frequency patterns. Within the vision-based direction controller this results in a reduced weighting of nearnesses obtained from marginally textured objects (i.e. white walls) when computing the collision avoidance direction *CAD*_*fov*_ [Eq (6)]. Hence, the controller’s preference for routes along certain boundaries of the experimental arena is a result of the inconsistent distribution of spatial frequency and contrast within the environment (see Figs 7 and 9D).

## Evaluation of power consumption and resource efficiency

The vision-based direction controller used for collision avoidance and navigation on the hexapod walking robot HECTOR is based upon computational mechanisms found in the visual pathway of insects. Since the biological and the technical substrate of the respective computational system are inherently different, a direct comparison between the two is not possible. Therefore, this project concentrates on a resource efficient implementation of the bio-inspired algorithms on a suitable hardware. This raises the question which hardware approach is suitable to a) host the bio-inspired vision processing algorithms and b) reach a level of efficiency during vision processing that—as compared to concurrent technical implementations—is optimal w.r.t. number of frames per second (application of the complete vision algorithm for each frame) related to invested power. In order to compare the resource efficiency of the FPGA-based implementation (Apalis module with Zynq SoC) used on HECTOR two additional hardware implementations were tested. These implementations comprise a serialized CPU-based design, as well as a parallelized design on the embedded graphics processor (GPU) of an Apalis module with a highly energy-efficient SoC that also can be directly integrated in HECTOR. It will be shown that the FPGA-based implementation is more efficient than the implementations based on either CPU or GPU processing. For the direct comparison of the different implementations and the simulation results, a resolution of 8 bit per pixel is chosen. In this mode, the bits [9:2] from the 10 bit ADC are transmitted and therefore the dynamic range of the camera decreases merely by 5.87% compared to the 10 bit per pixel transmission. The two following sections focus on the use of FPGA resources (device utilization) and the power consumption of the implementation on the Zynq SoC. The final section compares the efficiency of the three designs.

### Resource consumption of the FPGA-based SoC implementation

The first implementation of the proposed bio-inspired vision processing algorithm uses the novel Apalis Zynq module which is based on a Xilinx Zynq XC7Z020 SoC. The FPGA device utilization of the proposed design is shown in Table 1. The architecture needs 39.35% of the slices and 35.71% of the internal 36KB BRAMs for the processing of camera images with a downscaled resolution of 128 x 128 pixels (comparable with the low resolution in insect vision) after the first processing step (*ReMap* core, see Fig 4). An extension of the pixels to 10 bit resolution in the proposed FPGA design results in an increase of the required resources: slices by 3.48%, internal 36kB BRAMs by 4.29% and DSPs by 1.82%.

**Table 1:** Total FPGA resources for bio-inspired processing.

Zynq XC7Z020	Slices	FF	LUT	BRAM (36K)	DSP48	BUFG
Max. Amount	13300	106400	53200	140	220	32
Complete Design	5233	16666	11700	50	16	5
Percentage	39.35%	15.66%	22.12%	35.71%	7.27%	15.63%

The total amount of resources required for the FPGA-based vision processing is presented in Table 2. The overall number of slices needed for the insect-inspired processing amounts to 1342, which corresponds to 10% of the total number of the available slices within the Zynq SoC. 31 BRAMs (36KB) and 4 DSP48s are used for building the individual processing cores. Most of the logic resources are used in the *SA* and *EMD* cores. The *ReMap* core uses most of the BRAMs with an amount of 17.5 followed by the *HPF* and *LPF* cores with 8.5 and 4.5 BRAMs. The size of the memories within the cores is resolution dependent and scales linearly with the image size. The DSPs are used for the elementary motion detectors (*EMD* core) and the following calculation of the motion energy (*ME* core).

**Table 2:** FPGA IP-core resources for bio-inspired processing.

IP-Cores	Slices	FF	LUT	Logic LUT	Memory LUT	BRAM (36K)	DSP48
ReMap	122	389	239	232	7	17.5	0
SA	319	835	426	404	22	0.5	0
HPF	189	169	132	130	2	8.5	0
LPF	142	194	128	125	3	4.5	0
h / v EMD	341	949	681	586	95	0	2
ME	130	516	732	671	61	0	2
ANV	99	225	208	132	76	0	0
**SUM**	1342	3277	2546	2280	266	31	4

### Comparison of the FPGA-based design with CPU- and GPU-based designs

In order to compare the efficiency of the FPGA-based implementation of the vision-based direction controller on the Zynq SoC, two software implementations based on an Apalis CoM with Exynos SoC [44] have been tested. The Samsung Exynos 5250 architecture used on the CoM comprises an ARM Cortex-A15 Dual Core and a Mali T604 graphics processor; it is an SoC used in smartphones, which is highly optimized towards energy-efficient processing. The vision-based direction controller was implemented in a) an optimized CPU implementation as well as b) a parallelized design using OpenCL on the Mali-GPU of the Exynos SoC. For the benchmark of the FPGA-based design and the two software implementations the time to process a single frame (ms/frame) as well as the power dissipation was considered. The efficiency of the different designs was estimated by computing the frame rate to power ratio. Table 3 summarizes the benchmark results.

**Table 3:** Comparison of the software and FPGA-based hardware implementation.

Processing System	Processing Time [ms]	Frame rate [fps]	Power (Processing) [W]	Efficiency [fpJ]
Exynos CPU	13.519	74	0.200	370
Exynos GPU	0.792	1262	2.050	615.6
Zynq FPGA	0.1	10000	0.136	73529.4

The benchmark results show a significant reduction in processing time when comparing the serialized CPU-based implementation (13.519 ms, corresponds to 74 fps) with the parallelized GPU-based implementation (0.792 ms, corresponds to 1262 fps) of the vision-based direction controller on the Exynos SoC. By implementing the control architecture on the FPGA a further significant reduction of the processing time to 0.1 ms (10000 fps) has been achieved. The FPGA implementation uses the data types unsigned, signed and integer as data format while the CPU and GPU implementation use the data type floating-point in addition to the data type integer. This difference affects the accuracy of the computed data. The division within the temporary filter stages HPF/LPF in the FPGA is implemented by shift operations and subsequent additions. The IP core for the calculation of the motion energy (ME) uses the Xilinx CORDIC IP to implement the root square function. Here, the datatype unsigned integer is used. For the FPGA implementation, bit widths of the integer operations have been chosen to minimize the possible error, hence, leading to accuracy comparable to the CPU/GPU implementation. Simulations have shown that the results achieved with the integer calculations deliver a very accurate mapping of the desired bio-inspired application. Both, the Exynos as well as the Zynq CoM module require approx. 3.5 W in idle when using a Linux-based operating system [45]. This base power measured directly on the modules is mainly required for the highly flexible power distribution and networking interfaces. In future optimized versions of the hardware, base power can be significantly reduced by just supporting the interfaces required in the used robot platform. Therefore, our main optimization goal is the additional power that is required when running the vision-based direction controller, which amounts to 0.2 W for the CPU-based Exynos implementation and 2.05 W for the GPU-based design. For the FPGA-based implementation, although providing the highest performance, the power consumption increases just by 0.136 W compared to the base implementation. The total power dissipation is determined by measuring the supply voltage and the current consumption of the complete module. The power consumptions of the individual CPU and GPU software implementations are determined by the difference of the measured module power dissipation for the different operating states. By contrast, the power dissipations of the individual FPGA-based IP-Cores are determined by applying the Vivado Power Analysis and the Xilinx Power Estimator (XPE) tools.

**Fig 10:**
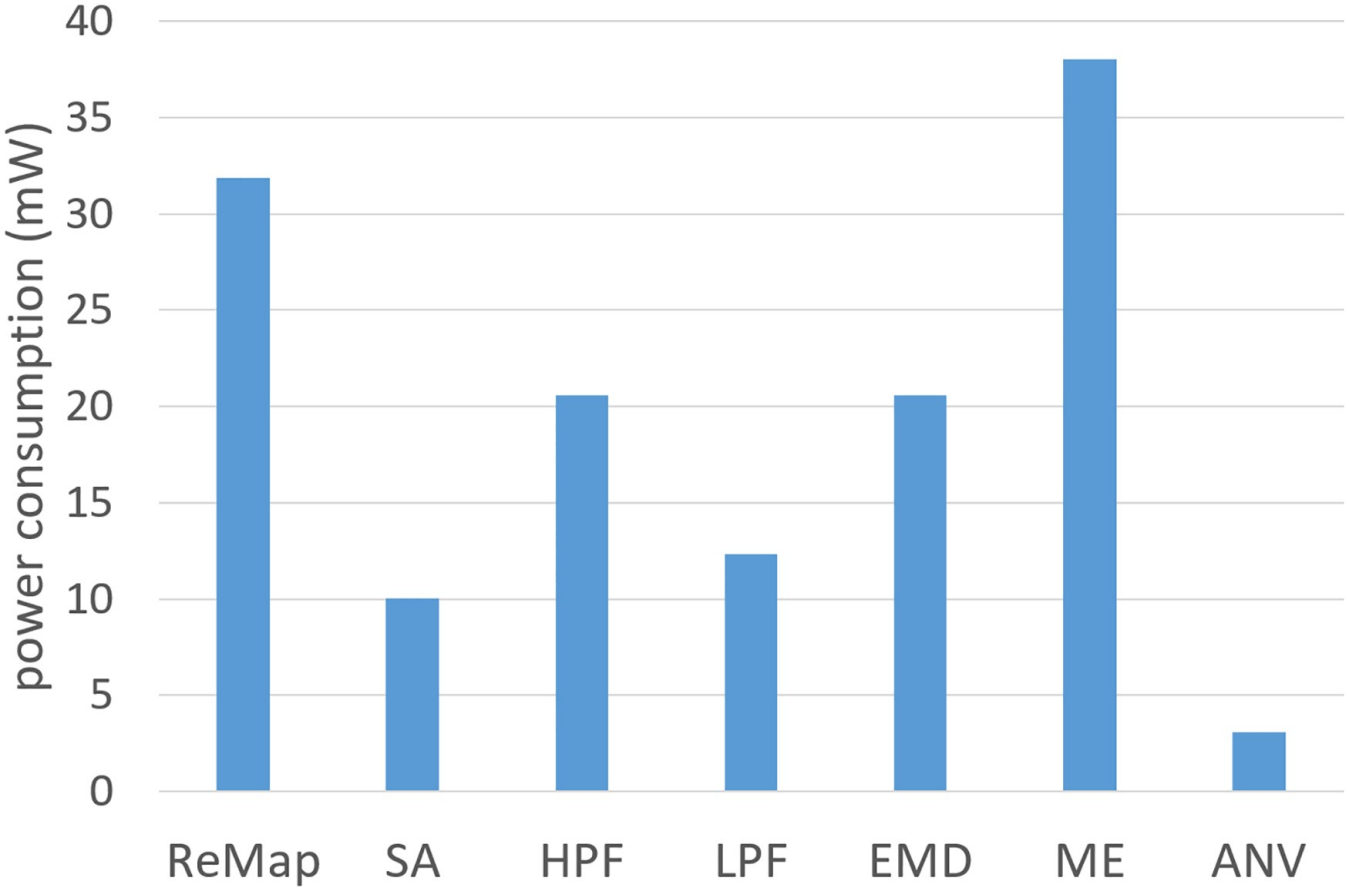
Power dissipation for the implementation of the vision-based direction controller on the Zynq SoC. Power consumption of the IP-cores implemented on FPGA (Explanation of abbreviations: ReMap: Remapping and downscaling; SA: Sensivity Adaption; HPF: High-Pass Filter; LPF: Low-Pass Filter; EMD: Elementary Motion Detector; ME: Motion Energy; ANV: Average Nearness Vector).

The individual power needed for the single processing cores implemented on the FPGA is shown in Fig 10. The most power is consumed by the motion energy core (*ME*) and amounts to 38.04 mW, which is only 1.73% of the overall power consumption of the Zynq XC7Z020 SoC (2.2W). The *ANV* core requires the least power with merely 3.08 mW (0.14%). The power needed for the FPGA-based bio-inspired visual processing cores corresponds to 6.19% of the overall power consumption of the Zynq SoC.

The non-accelerated solution on the Exynos CPU has the second lowest power consumption and the highest calculation time. Once the embedded graphics processor is used for the calculation, the power consumption of the Exynos module increases strongly, but it achieves significantly lower processing times. Due to the massive parallelization on the FPGA, a significantly faster processing than on the graphics processor is achieved combined with the lowest power consumption of the vision-based direction controller (in total as well as for the incremental part). The hardware implementation has an efficiency of 73529.4 fpJ (frames per Joule), which is nearly 119 times more than the 615 fpJ of the GPU solution on the Exynos SoC. The lowest energy efficiency is achieved using the serialized processing on the ARM processor of the Exynos with 370 fpJ. Correlating the inverse of the energy efficiency with the frame rate of 20 fps and the resolution of 128 x 30 pixels results in an energy per pixel of 14.1 *μ*J/pixel for the CPU and 8.5 *μ*J/pixel for the GPU at 20Hz. The FPGA implementation uses the lowest energy per pixel with 70.8 nJ/pixel at 20Hz. Thus, the hardware implementation on the FPGA is most energy efficient for the implementation of the vision-based direction controller.

## Conclusion

Compared to man-made machines animals show a remarkable behavioral performance in many respects. Although there has been tremendous progress in building mobile autonomous robots in the last decades, even insects outperform current robotic systems in terms of computational and energy efficiency, speed and robustness in different environmental contexts. Hence, from an engineer’s perspective the evolutionary optimized mechanisms underlying sensory processing and behavioral control in biological systems are potentially of great interest (for a recent overview see [46]). In this paper, an insect-inspired vision-based navigation and collision avoidance controller [16] was implemented on a novel embedded hardware module based on the Xilinx Zynq SoC. The module is based on the Apalis CoM (computer on module) standard and has been specifically designed for the highly efficient bio-inspired processing of visual information on autonomous robots. By leveraging a combination of optimized parallel processing on FPGA, serialized computing on CPU and direct communication without additional protocol overhead, different mechanisms found in the visual pathway of flying insects have been adapted to control the collision avoidance behavior of the stick insect-like walking robot HECTOR in a highly resource-efficient way. After optimization of the parameters of the vision-based direction controller in simulation, the relative nearness information obtained from optic flow estimation via EMDs is sufficient to direct HECTOR to a goal location in a real-world scenario without colliding with obstacles. In a recent study, the vision-based direction controller described in this paper was also tested in computer simulations of the same and two other scenarios [16]. The other scenarios consisted of a) an artificially created arena with 30 randomly placed objects and b) a scenario reconstructed from 3D laser scans of a meadow with trees. In all scenarios HECTOR was able to navigate to the target location without colliding with obstacles from different starting positions and using the same set of parameters obtained from the optimization. However, in different environments—e.g. in outdoor scenarios with dynamic lighting conditions—the generalizability of a certain parameter set might no longer be given. The bio-inspired walking controller of HECTOR [37] allows the robot to robustly navigate even in difficult terrain [10]. The FPGA-based implementation of the control architecture shows a drastic increase in performance and energy efficiency when compared to CPU- or GPU-based software implementation. This demonstrates the potential of the Apalis Zynq CoM presented here for the implementation and analysis of new bio-inspired vision processing algorithms. Due to the high frame rate, low weight and low energy consumption the module is ideally suited to be placed on fast moving robotic platforms, such as flying drones, where size or weight are limiting factors. In comparison to other recently proposed hardware solutions for bio-inspired processing of visual information on autonomous robots [47], the direct implementation of the processing unit on the robot avoids computational bottlenecks such as the transmission-related reduction of the frame rate. By employing behavioral strategies such as active head stabilization—which is also found in insects—it might be possible to further reduce the influence of rotational optic flow components which potentially obfuscate the estimation of relative nearness from optic flow [15]. Hence, a prototype for mechanical gaze-stabilization will be implemented on the robot in order to increase the collision avoidance performance of the vision-based direction controller.
